# Assessment of Supercritical CO_2_ Extraction as a Method for Plastic Waste Decontamination

**DOI:** 10.3390/polym12061347

**Published:** 2020-06-15

**Authors:** Ayah Alassali, Noor Aboud, Kerstin Kuchta, Philip Jaeger, Ahmad Zeinolebadi

**Affiliations:** 1 Sustainable Resource and Waste Management, TUHH—Hamburg University of Technology, Blohmstr., 15, 21079 Hamburg, Germany; noor.aboud@tuhh.de (N.A.); kuchta@tuhh.de (K.K.); 2 Eurotechnica GmbH, An den Stücken 55, D-22941 Bargteheide, Germany; philip.jaeger@eurotechnica.de; 3 Polymer Consult Buchner GmbH, Dorfgrund 6, D-22397 Hamburg, Germany

**Keywords:** plastic waste, decontamination, supercritical extraction, polycyclic aromatic hydrocarbons

## Abstract

Due to the lack of advanced methods to clean plastic waste from organic contaminants, this study aimed at evaluating supercritical extraction as a decontamination method. Oil-adhesive high-density polyethylene (HD-PE) oil containers were subjected to supercritical extraction using supercritical carbon dioxide. The extraction was conducted at 300 bar, applying various temperatures (i.e., 70, 80 and 90 °C). The study assessed the impact of temperature on the decontamination efficiency. The variation in the samples’ quality was first analyzed using near infrared (NIR) spectroscopy. An analysis of the content of polycyclic aromatic hydrocarbons (PAHs) was followed. Samples treated at 70 and 80 °C showed higher extraction efficiencies, in spite of the lower extraction temperatures. The NIR analysis showed that the plastic specimens did not experience degradation by the supercritical decontamination method. Moreover, the NIR spectra of the extracted oil showed the presence of a wide range of compounds, some of which are hazardous. This has been confirmed by a GC-MS analysis of the extracted oil. Based on the provided assessment, the quality of the decontaminated HD-PE plastic samples—from a contamination point of view—is enhanced in comparison to untreated samples. The level of PAHs contamination decreased to be within the allowed limits defined by the REACH regulation, and also met the specifications of the German Product Safety Committee. This study proved the effectiveness of the supercritical extraction using CO_2_ in extracting organic contaminants from plastics, while maintaining their quality.

## 1. Introduction

The total post-consumer plastic waste generated in Europe and Turkey combined in 2018 summed to 42 million metric tons (Mt) [1], representing ~17% of the globally produced plastic waste. Germany alone produced 5.2 Mt of post-consumer plastic waste in 2017, whereas only ~16% of the post-consumer plastic waste followed a closed-loop recycling process [2]. On the other hand, Germany was the third top country in plastic waste exports in 2018, following the USA and Japan. About 53% of all exported plastic waste is sent to Asian countries [3]. However, China recently banned the import of plastic waste [4,5]. In response, the European Commission communicated that by 2030 all plastic packaging will be recyclable on the EU market [6]. Furthermore, the EU set specific recycling targets for plastic packaging to be 50% from 2025 onwards and 55% from 2030 onwards [7]. Consequently, the closed-loop recycling of plastics has to be broadened, and robust domestic recovery and recycling solutions have to be developed and implemented.

One main challenge in plastic recycling is the heterogeneity of the plastic waste streams, containing a wide-range of polymers, polymer grades, chemical additives, and contaminants [8]. Particularly, contamination hinders the reuse of recycled plastics in several applications, such as the food and beverage industry. Contamination takes place through all phases of the value chain, starting from the polymer production (additives), the manufacturing step (e.g., labels, coloring and inks), during service (mostly non-polymer impurities as well as degradation products), and finally during plastic waste collection, and sorting (polymer cross contamination) [9].

Thus, the risk of the presence of undesirable chemicals that can migrate and accumulate in the plastic cycle poses a significant challenge to the closed-loop recycling of plastics [10]. Among the different chemicals, polycyclic aromatic hydrocarbons (PAHs) particularly complicate the closed-loop recycling of plastics. Although there are thousands of PAH compounds, most studies consider 16 PAHs, those specified by the U.S Environmental Protection Agency (EPA) [11]. PAHs are environmental pollutants and possess toxic, carcinogenic and mutagenic characteristics [12,13]. Many of the PAH substances are categorized as persistent, bio-accumulative and toxic (PBT) substances. These substances do not degrade and can remain for extended periods in the environment [12]. Polycyclic aromatic hydrocarbons can be found in plastics, primarily as a result of the contamination of the raw materials used for plastics’ production, by the additives added during the manufacturing stage (by design), and/or as a result of contamination by application (environmental pollutant) [14,15].

The European Commission published the Commission Regulation (EU) No 1272/2013 of 6 December 2013 amending Annex XVII to Regulation (EC) No 1907/2006 of the European Parliament and of the Council on the Registration, Evaluation, Authorization and Restriction of Chemicals (REACH) as regards polycyclic aromatic hydrocarbons [16]. The regulation bans articles containing rubber or plastic fractions with certain levels of PAHs from being placed on the market for sale to the general public in the European Union. This amendment considers plastic and/or rubber articles, which may come into direct and persistent contact or short-term, yet repetitive contact with human skin or mouth. The articles that fall under this regulation include various products of different applications (e.g., sports gear, household tools and equipment, clothing and accessories). The regulation declares that the defined articles cannot contain more than 1000 µg kg^−1^ (0.0001% per weight) of the 8 selected PAHs (priority PAHs). On the other hand, toys or children’s articles have a restriction limit of 500 µg kg^−1^ (0.00005%) [17]. In May 2019, the German Product Safety Committee (Ausschuss für Produktsicherheit, AfPS) issued new specifications for PAHs under the voluntary ‘Tested Safety’ Mark (Geprüfte Sicherheit Mark (GS-Mark)). These specifications will become effective on 1 July 2020 [18].

There have been several research activities on decontamination of different polymeric matrices using supercritical fluid extraction (SFE) [19,20,21,22]. The previous studies proved the effectiveness of the SFE in extracting different organic contaminants, such as ammonium carboxylate perfluoropolyether surfactants [23], citrate and benzoate plasticizers [24], antioxidant additives [25], and stearin, dilaurin, trilaurin, and tripalmitin [25]. Supercritical fluid extraction is a separation process, where the substances are dissolved in a fluid in its supercritical state. Supercritical fluid extraction is used for separation, purification and concentration purposes [26]. In the supercritical state, the physicochemical properties strongly depend on the applied pressure and temperature, for which the capacity to dissolve compounds is easily tunable by carrying these process parameters [27]. The supercritical fluid extraction has a large number of applications in the food, pharmaceutical and cosmetic industries, especially as a technology to extract and produce bioactive compounds at high quality [28,29].

The utilization of supercritical fluids in industrial processes has environmental advantages for working with non-hazardous fluids like CO2 in a closed cycle, and therefore potentially replacing conventional organic solvents that are more environmentally damaging. However, the high investment costs of supercritical fluid extraction is considered as the main disadvantage of its application [29]. Current developments in semi-continuous processing or multi-vessel extraction attempt to overcome this drawback [30].

Among the available supercritical fluids (SCFs), supercritical CO_2_ (SC‒CO_2_) is the most used fluid due to its beneficial thermodynamic and transport properties, as well as its environmental impact, as it was approved by the U.S. Food and Drug Administration (FDA) and the European Food Safety Authority (EFSA) [31]. Carbon dioxide is low cost, non-corrosive, non-explosive, easy to handle, non-reactive, non-flammable and non-toxic. Additionally, it could be collected from industrial processes and reused in a cycle. The most essential thermodynamic effect of the CO_2_ is the tunable solvation power close to and above the critical point [32]. The solvation power of CO_2_ is strongly dependent on the applied pressure. Generally, SC‒CO_2_ has a mild supercritical temperature (31.1 °C) and pressure (1071 psi), thus it is convenient to separate heat-labile substances. It also offers a small and linear molecular structure, which causes a faster diffusivity, resulting in an efficient mass transfer, in addition to the low viscosity that facilitates its penetration into the solid matrix during the extraction process. The density of the supercritical CO_2_ is relatively high, which indicates that its solvation power is higher than many other SCFs [32,33]. Furthermore, using SC‒CO_2_ will leave no solvent residues in the extract and thus will contaminate neither the extract nor the remaining extracted solid [32].

In this research, supercritical extraction (SCE) was utilized as a decontamination method for plastics originating from oil canisters. The canisters—made of high-density polyethylene (HDPE)—have been in contact with hydrocarbon and lubricant oils, which are difficult to clean by available washing processes because these hydrocarbons may have partly infiltrated the plastic. The extraction is conducted at a constant pressure (300 bar), while the temperature was varied to assess the impact of the temperature on the extraction efficiency and quality. Generally, the pressure has a great impact on the solubility, whereas the temperature affects the CO_2_ diffusivity. The effectiveness of the extraction was assessed by performing a PAH-content analysis.

## 2. Materials and Methods

### 2.1. Plastics Decontamination Applying Supercritical Extraction

The test sample originated from high-density polyethylene (HDPE) containers, which were in contact with hydrocarbon oils and lubricants. The containers—provided by Brockmann Recycling GmbH, Germany—were shredded to flakes in a size range of 2 to 4 cm.

The supercritical extraction procedure was conducted using a 4 L high-pressure extraction unit HPE lab 700r (Eurotechnica GmbH, Bargteheide, Germany) (P_max_ = 70 MPa, T_max_ = 150 °C). The equipment was described elsewhere [34]. Carbon dioxide was provided by Westfalen AG, Muenster, Germany, at a purity of 99.999 Mol-%.

**Figure 1:**
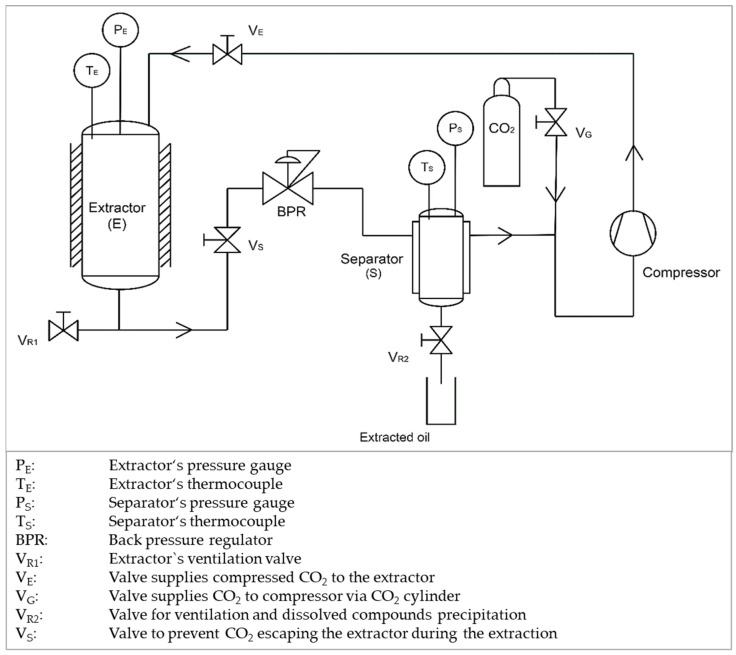
A schematic presentation of the supercritical extraction (SCE) system utilized in this experiment.

Heating of the extraction and the separation vessels is carried out by means of a heating circulation system—SE26 and SE12 MA by Julabo (Seelbach, Germany) (maximum allowable working temperature 150 °C). In order to compress the CO_2_ gas to the desired pressure, an electrical driven membrane gas compressor (Nova Swiss, Effretikon, Switzerland) is employed. The compressed CO_2_ enters the extraction vessel (P_max_ = 69 MPa, T_max_ = 150 °C, internal volume 4 L) at the top and flows through the vessel in a top to bottom fashion. At the outlet, the extraction pressure is controlled by means of a manual backpressure regulator (BPR, Tescom Europe, Selmsdorf, Germany), with a maximum allowable working pressure of 69 MPa, at a temperature of 80 °C and a maximum volume flow of 5 kg h^−1^. To precipitate the dissolved compounds obtained during the extraction process, a separation vessel (P_max_ = 50 MPa, T_max_ = 100 °C, internal volume 0.5 L) is operated at a pressure not far from the cylinder pressure. After separating the extract, the recovered CO_2_ is conducted to the suction inlet of the compressor for recompression and operation in a closed loop. The operation temperature in the extractor and the separator are recorded using calibrated thermocouples (accuracy of ±0.1 °C), while the pressure is recorded by calibrated pressure gauges (accuracy of ±5 bar). A schematic presentation of the extraction unit is shown in Figure 1.

The series of experiments was divided into three batches, B1, B2 and B3. The applied pressure in the three batches was identical (300 bar), while the temperature was varied: 70, 80 and 90 °C.

The plastic samples were weighed before and after extraction. A product basket containing a sintered metal bottom was used to place the source material for the extraction. This basket was closed by a filter in order to prevent blockage of tubing by entrained particles.

### 2.2. Plastic Specimens’ Characterization

#### 2.2.1. Near Infrared Spectroscopy (NIR) Analysis

The NIR spectroscopy analysis was conducted using Bruker (Billerica, MA, USA) Optics FT-NIR spectrometer MPA (Multi-Purpose Analyzer). A triple scanning was conducted on each of the tested samples, each containing 256 scans with a resolution of 16 cm^−1^. The measurements were obtained in absorbance units.

#### 2.2.2. Degree of Plastic Contamination with Polycyclic Aromatic Hydrocarbons (PAHs)

The degree of plastic contamination with PAHs was analyzed, to evaluate the efficiency of the extraction method. The procedure is described below. The test considered the original sample (untreated), and samples from B1 and B2.

- PAHs extraction by Randall hot extraction
  Test samples underwent preparation by extraction using Randall hot extraction. The applied extraction method was adapted from the method suggested by Geiss et al., 2018 [35]. Since the extraction temperature was not provided, a temperature slightly higher than the solvent’s boiling point was first applied and revised as needed.
  First, the samples were weighed in duplicates in 33 × 60 cellulose extraction sleeves (thimbles). The weight of the test samples ranged between 0.1 and 0.2 g. The reaction vessel, in which the extract is collected, was filled with 70 mL of toluene, and three to four boiling stones were added before connecting it to the condenser.
  Randall hot extraction consists of three main steps, immersion, rinsing and evaporation. The immersion step was conducted at 150 °C for 120 min. Subsequently, the thimble was pulled out of the sump (reaction vessel containing the extraction solvent). At this stage, the temperature was raised to 170 °C to rinse the sample for 60 min. The evaporation step was conducted at the same temperature (i.e., 170 °C) for as long as needed to reach a solution’s volume of ≤20 mL.
  After reaching the targeted extract volume, the heating was turned off and the reaction vessels were cooled down to room temperature. Then, 40 μL (Conc. = 40 ng mL^−1^) of an internal standard solution (Phenanthrene-d10 98 atom % D, 98% (CP) from Sigma-Aldrich (St. Louis, MO, USA)) was added to the solution to calculate the recovery. The extract was then filtered into a 100 mL pear-shaped flask. Finally, the extract was evaporated by a 0.7 bar nitrogen (N_2_) stream at room temperature. Drying was applied until the solution reached a volume of ~500 μL.
- Extract clean-up
  To selectively extract polycyclic aromatic hydrocarbons (PAHs), a clean-up procedure was applied. The extract clean-up was achieved by applying solid phase extraction (SPE), by means of silica gel disposable extraction columns. The procedure of SPE consisted of column cleaning, silica bed conditioning, sample application, and elution. For silica bed conditioning, 5 mL dried n-Hexane were added to the SPE column twice. For sample elution (to separate the PAHs), 2 mL of dried CH_2_CL_2_/Hexane mixture was added to the column. This step was repeated twice in series and the eluate was collected in clean tubes. Finally, the obtained solution was concentrated to 500 μL using a 0.5 bar N_2_ stream.
  To prepare the test sample for the GC-MS analysis, the concentrated sample was transferred using a Pasteur pipette to a 1.0 mL brown glass GC-vial. The final sample volume was brought to 1.0 mL by adding n-Hexane.
- Analysis by GC-MS
  The analysis of the samples was performed using a gas chromatograph HP 6890 coupled with a single ion-monitoring mass selective detector HP 5973 from Agilent Technologies Sales & Services GmbH & Co. KG (Waldbronn, Germany).
  Before conducting the analysis, a calibration step was conducted. The calibration was done prior to analyzing the sample set. For the calibration, different established concentrations of an external standard containing the 16 US-EPA PAHs—naphthalene, acenaphthylene, acenaphthene, fluorene, phenanthrene, anthracene, fluoranthene, pyrene, benzo[a]anthracene, chrysene, benzo[b]fluoranthene, benzo[k]fluoranthene, benzo[a]pyrene, indeno[1,2,3-c,d]pyrene, dibenz[a,h]anthracene, and benzo[g,h,i]perylene—were analyzed, and the respective responses were measured.

### 2.3. Extracted Oil Characterization

#### 2.3.1. GC-MS Analysis of the Extracted Oil

The oil samples were first prepared by homogenization and consecutive heating at 50 °C in a drying oven. About 0.5 g was weighed into a 5 mL volumetric flask, where the sample was dissolved in n-Hexane. Afterwards, each sample was dried with sodium sulfate (NaSO_4_) and finally centrifuged. Gas chromatograph (GC) was performed using a column of Agilent DB (30 m × 0.25 µL ID) at 40 °C for 1 min. At a rate of 15 °C min^−1^, the temperature was raised to 320 °C for 25 min. The total run time was 44.7 min. The mass spectrometry (MS) was performed using a normal scan with one scan rate of about 12 scan s^−1^. The database from the National Institute of Standards and Technology (NIST) was used for matching the analysis results.

#### 2.3.2. Near Infrared Spectroscopy (NIR) Analysis

The abovementioned NIR analysis method was conducted on the extracted oils to identify the chemical composition.

## 3. Results and Discussion

### 3.1. Assessment of the Extraction Behavior

**Figure 2:**
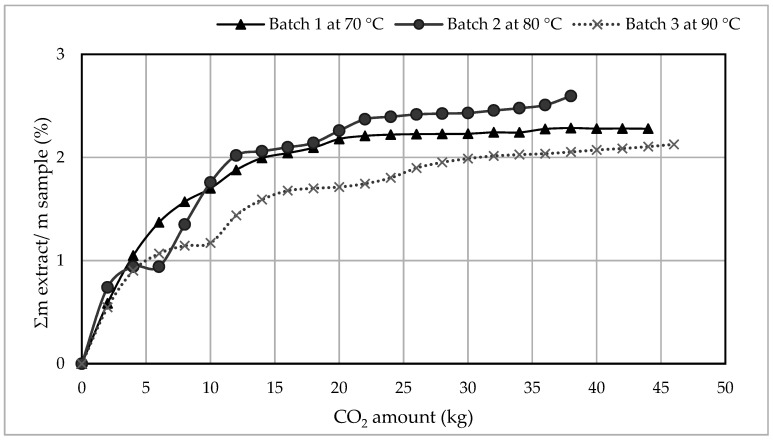
A Comparison of the cumulative SCE rates for each of the test batches in relation to the amount of the used SC-CO_2_.

The supercritical CO_2_ extraction was executed on three different batches, B1, B2, and B3, where the extraction temperatures were 70, 80, and 90 °C, respectively. The extraction kinetics was determined by quantifying the extract and calculating the cumulative extraction yield in relation to the total supercritical CO_2_ used for extraction (see Figure 2). The cumulative extraction yield is calculated by dividing the cumulative extract’s mass by the mass of the original sample put for extraction.

The experiment was terminated when no further oil extraction was obtained (constant weight). The total operation (extraction) time was 22, 19 and 23 h for B1, B2 and B3, respectively. With a CO_2_ flow rate of 2 kg h^−1^ (the flow rate applied in the extraction), the total supercritical CO_2_ consumed was 44, 38 and 46 kg for B1, B2 and B3, respectively.

The extraction rates indicated that the highest increase in the extraction yield was obtained in the first five hours of extraction, using 10 kg of CO_2_ (see Figure 2). The extraction yield increased slightly for B1 and B2 by increasing the amount of the extraction solvent from 10 kg to 20 kg, after which a constant extract weight was maintained (no further extraction). In B3, the increase continued up until the end of the extraction, however, the increase was insignificant. During the first hours of the SCE, the extraction was obtained from the surface of the plastic flakes. Hence, the extraction rate was the highest. Later, at longer soaking periods, the supercritical CO_2_ diffused into the pores of the polymers, extracting the minor quantities migrating into the polymeric chains.

Moreover, comparing the extraction kinetics for the three batches showed that the extraction rate in the first three hours was similar in the three test batches. After the first three extraction hours, differences in the extraction kinetics were obtained. Hence, the impact of changing the temperature was only seen after the first three hours of extraction.

Although the extraction temperature in B3 was the highest, the extraction rate was the lowest. This indicates that increasing the temperature beyond a certain point affects the fluid’s density (the density decreases with an increase in the temperature) more strongly than the volatility of the extracted compounds. This result is similar to what was reported by other studies [33,36], where a crossover point was observed. The crossover pressure can be defined as the point where the slope of the plot of solubility versus temperature changes sign [37]. Below this point, the solubility decreases with the increasing of the applied temperature under isobaric conditions. Above the crossover point, the solubility increases with the increasing of the applied temperature. Apparently, the pressure in this work is very close to the crossover point, for which there is an optimum temperature.

Due to the higher extraction yields from B1 and B2, samples from these two batches were subjected to further analyses along with the original sample (before extraction).

### 3.2. Evaluation of the Samples Before and After Undergoing SCE

#### 3.2.1. General Analysis

The samples were first analyzed visually, the color and appearance of the specimens changed after applying the supercritical extraction for the three batches. It can be observed how the polymers lost their glossy appearance after undergoing SCE, indicating the effectiveness of the SCE in removing surface dirt (oil). In addition, the original sample had a distinctive smell of hydrocarbons, which was absent in extracted samples.

CO_2_ is gaseous at room temperature and atmospheric pressure. Hence, the samples became free of solvents under ambient conditions. This was remarked by comparing the weights of the samples immediately after SCE with their weights after few hours, where weight decreased by 1.30%, 1.39%, and 1.63%, for B1 and B2 and B3, respectively. Generally, the weight increase in the samples due to CO_2_ diffusion increased by increasing the operation temperature. Hence, the higher the extraction temperature, the higher the immersion of the CO_2_ into the plastic samples. Although the highest CO_2_ was obtained in B3, this batch provided the lowest extraction yield. This indicates the domination of the effect of the fluid’s density rather than its diffusivity.

#### 3.2.2. NIR Analysis

**Figure 3:**
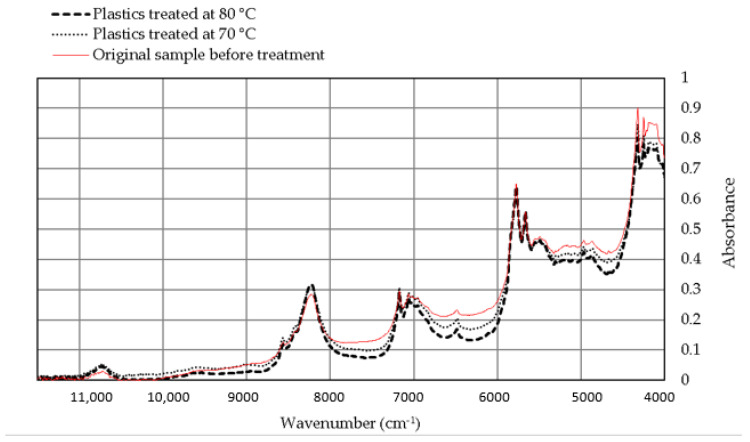
The near infrared (NIR) spectra of high-density polyethylene (HD-PE) samples before and after undergoing SCE.

The NIR analysis of specimens before and after extraction was similar in terms of trend and absorbance units (see Figure 3).

Slightly higher absorbance intensity was observed in untreated plastics. This is attributed to the darker color of this material due to its contamination with oils.

The treated HD-PE specimens showed a slight increase in the absorbance units at wavenumber 10,730 cm^−1^ (O-H, water/moisture) [38], which could be increased by the decreased hydrophobicity of the samples after extraction, and hence the higher moisture content. An increase in the absorbance units was also seen at 8960 cm^−1^ (C═C alkene, vinyl group, second overtone), and 8250 cm^−1^ (CH_2_ methylene, second overtone of symmetrical vibration) [39]. On the other hand, the treated samples had lower absorbance units at 5600 cm^−1^ (CH_3_ first overtone of symmetrical vibration). This slight change in the absorbance units for the mentioned peaks (8960, 8250 and 5600 cm^−1^) could have happened through the chemical bonding of carbon dioxide during the SCE.

The change in the spectra after sample treatment is too minor to indicate any formation of new chemical groups (oxidative groups). Hence, the application of SCE on HD-PE did not result in sample degradation.

#### 3.2.3. Efficiency of Plastics Decontamination, Focusing on the PAHs Content

Polycyclic aromatic hydrocarbons (PAHs) are organic compounds made of benzene rings; usually consisting of 2 to 7 benzene rings formed in linear, angular, or cluster arrangements [40]. PAHs can be produced during crude oil maturation and associated processes. Hence, PAHs can be found in products derived from petroleum. When PAHs are not removed from refinery products, they enter the environment due to their persistence [12,41].

**Figure 4:**
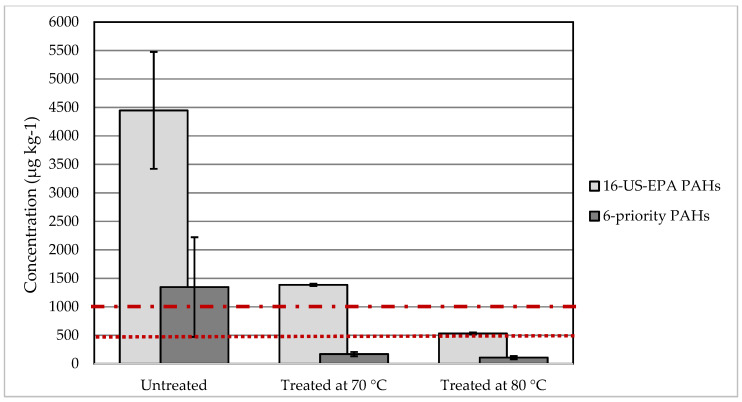
The sum of the 16-US-EPA polycyclic aromatic hydrocarbons (PAHs) content and the 6-priority PAHs in samples before and after pretreatment by SCE. The dotted lines represent the REACH limits for the 8 priority PAHs in consumer products (upper line) and children articles (lower line).

Analyzing the PAHs content in the HD-PE samples can define the applicability of the recyclates produced from them. The concentration of the single PAHs was obtained as per the GC-Ms analysis (explained in the materials and methods section). The total concentration of the 16-US-EPA PAHs and the concentration of the 6-priority PAHs were calculated for the samples’ duplicates and the average value and the standard deviation are presented in Figure 4. The analysis showed that untreated samples obtained concentrations of the 16-US-EPA PAHs that are three times and eight times higher than what was obtained for samples treated at 70 °C and at 80 °C, respectively. At 80 °C, plastic decontamination was more efficient, as could be seen from the total PAHs content. Among the 16 US-EPA PAHs, six belong to the eight priority PAHs (i.e., benzo[a]anthracene, chrysene, benzo[b]fluoranthene, benzo[k]flouranthene, benzo[a]pyrene, and dibenzo[a,h]anthracene). In this study, the total concentration of these six priority PAHs was analyzed, showing that plastics treatment by SCE could reduce the total content by 87% and by 92% for samples treated at 70 °C and 80 °C, respectively, in comparison to the original sample. The content of these 6-priority PAHs in the original sample (before treatment) did not comply with the REACH regulation, neither for children’s articles, nor for consumer products. Consequently, the quality of recyclates produced from this HD-PE sample without extraction treatment would be inappropriate for products on the European market. On the other hand, after treatment by SCE, the samples were decontaminated and the PAHs’ limits complied with the REACH threshold’s limits for children’s articles as well as consumer products (see Figure 4).

According to the specifications of PAHs under the voluntary ‘Tested Safety’ Mark (Geprüfte Sicherheit Mark (GS-Mark)) published by the German Product Safety Committee (Ausschuss für Produktsicherheit, AfPS), recyclates from the original sample (before treatment) are not applicable for products in category 1 or category 2 for children’s articles (under the age of 14) (see Table 1). This is due to the exceeding concentrations of benzo[a]anthracene, chrysene, and indeno[1‚2,3-cd]pyrene. The sample treated at 70 °C complied with the specifications for category 2 and category 3. However, it failed to comply with the specifications of category 1 due to the exceeding concentration of the sum of the 15 PAHs (see Table 1). Applying supercritical extraction at 80 °C on plastics contaminated with PAHs was efficient in decontaminating the samples, where the PAH concentrations decreased to comply with all three categories (defined by the German Product Safety Committee).

### 3.3. Analysis of the Extracted Oil

#### 3.3.1. Quantitative Analysis

Extracted oil was collected on an hourly basis. The initial sample weight put for extraction varies. Hence, for comparison purposes, the sum of the mass of extracted oil in each of the batches was divided by the initial mass of the sample in (wt%). The highest extraction yield was obtained for B2, achieved at 80 °C (2.7%), and the lowest was shown for B3, done at 90 °C (2.2%). This confirms that the effect of increasing the temperature beyond a certain point can result in a decrease in the supercritical fluid extraction efficiency.

#### 3.3.2. Qualitative Analysis

The extracted oil from the three extraction batches w similar in color (i.e., yellow brown liquid). The GC-MS spectra for oil from B2 (O80) had the same configuration as the GC-MS spectra for oil from B1 (O70) in terms of shape, but not in terms of count. The properties of the components and the molecular structures of the extracted oil are listed in Table 2. These components are considered highly toxic and hazardous to the environment and humans according to the Globally Harmonized System of Classification and Labeling of Chemicals (GHS). Therefore, the U.S. National Library of Medicine suggested that these components should neither come into direct contact with humans nor be discarded into the environment without treatment.

The extracted compounds can be reutilized in the form of fuel for energy recovery. In addition, these oils can also be utilized in the automotive industry, detergent manufacturing, fuel additives, and lubricants for engines, as well as brake fluids [42,43,44].

Oil extracted at 80 °C shows more presence of some compounds compared to the one extracted at 70 °C; except for Methoxytriethylenglycol. This can be attributed to the volatility of Methoxytriethylenglycol, which was higher at 80 °C.

**Figure 5:**
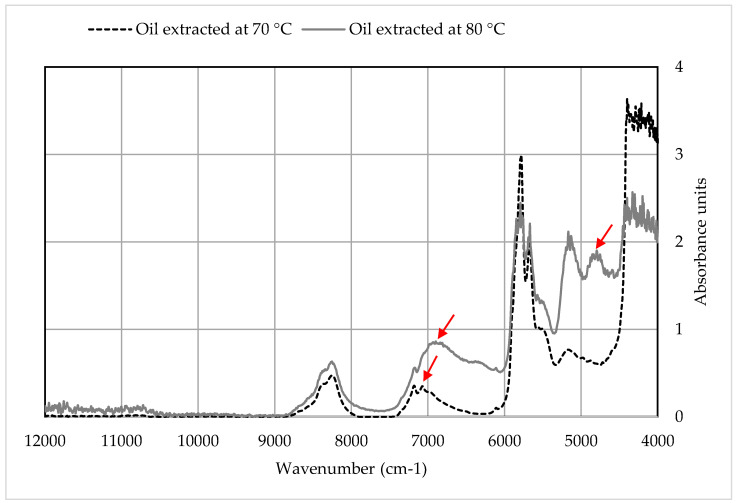
The NIR spectra of the extracted oil. The red arrows indicate the spectral shift between the oil extracted at 70 °C and the one extracted at 80 °C.

The extracted oils from B1 and B2 were analyzed by NIR spectroscopy (see Figure 5). The obtained spectra for oils from both batches showed a similar configuration, with minor differences recognized in the region 7000–4000 cm^−1^, and largely in the absorbance intensities. This indicated different concentrations of the extracted components. The NIR spectra of the oil extracted at 80 °C showed a shift in one peak in comparison to the oil extracted at 70 °C (from 6925 to 7060 cm^−1^). The former indicates a first overtone to an intermolecular hydrogen bond in −OH alcohol, while the latter shows OH-stretch and OH deformation in alcohol. Hence, the shift does not indicate differences in the composition, but differences in the vibrational modes.

Additionally, the NIR analysis of oil extracted at 70 °C showed a peak at wavenumber 5180 cm^−1^, which is representative for a second overtone for 2 × C═O stretching. On the other hand, the NIR analysis of the oil extracted at 80 °C exhibited a new peak at wavenumber 4800 cm^−1^ (indicative for −OH (alcohol)) in addition to the one at wavenumber 5180 cm^−1^. Accordingly, the C═O group in oil extracted at 70 °C is for ester, while the one in oil extracted at 80 °C is for carboxylic acid. This was recognized by the presence of both Ethylenhexanoic acid and (Butoxyethoxy) ethanol in higher concentrations in the oil extracted at 80 °C.

Overall, the oil extracted at 70 °C is similar to the one extracted at 80 °C, with different concentrations of the existing functional groups. This was confirmed by the results obtained by the GC-MS analysis. Hence, the applied temperature only affected the efficiency of extraction from a quantitative standpoint.

## 4. Conclusions

There is a general worldwide consensus that consumption of plastic must be reduced, and when not possible, its recycling must be enforced. Nevertheless, the contamination of plastics significantly limits the application of closed-loop recycling and reuse on the plastic waste stream. The aim of this research was to assess the supercritical extraction methodology using supercritical CO_2_ as a plastic waste decontamination method. The focus was on polycyclic aromatic hydrocarbons that are not removed by conventional washing procedures, and therefore accumulate in the plastic waste stream.

Supercritical extraction proved to be efficient in decontaminating plastic waste from organic contaminants. Specimens treated by supercritical extraction provided a quality complying with the REACH regulations with respect to the polycyclic hydrocarbon contents. This was valid for consumer products as well as for children’s articles. At the investigated extraction pressure, the extraction efficiency was determined by the applied temperature in the following way: A slight increase in the extraction yield was observed when raising the temperature from 70 °C to 80 °C. However, it dropped considerably at 90 °C, suggesting that the lower solvent power due to the lower CO_2_ density is the limiting factor in the extraction. The NIR analysis of plastic specimens before and after undergoing supercritical extraction did not show the formation of oxidation groups for which degradation can be excluded. Accordingly, supercritical extraction may be recommended for decontaminating high-quality and high-value plastics and therefore providing appropriate means to provide clean feedstocks for the following recycling steps. After all, the quality of recycled plastics is determined by the cleanliness of the recovered plastic feed. Hence, developing and optimizing efficient decontamination processes to treat plastic waste for recycling will help in achieving the closed-loop recycling quotas while providing high-quality and safe recyclates.

## Figures and Tables

**Table 1:** Maximum levels of PAHs allowed by the German Product Safety Committee [18].

Limits (µg kg^−1^)						Analyzed Samples		
Compound	Category 1	Category 2		Category 3		Original	Treated at 70 °C	Treated at 80 °C
		Children under 14	Other Products	Children under 14	Other Products			
Benzo[a]pyrene	<200	<200	<500	<500	<1000	113.4	20.2	13.0
Benzo[e]pyrene	<200	<200	<500	<500	<1000	n. a.	n. a.	n. a.
Benzo[a]anthracene	<200	<200	<500	<500	<1000	266.2	17.6	14.9
Benzo[b]fluoranthene	<200	<200	<500	<500	<1000	119.7	15.9	12.6
Benzo[j]fluoranthene	<200	<200	<500	<500	<1000	n. a.	n. a.	n. a.
Benzo[k]fluoranthene	<200	<200	<500	<500	<1000	64.6	11.4	8.4
Chrysene	<200	<200	<500	<500	<1000	419.1	93.2	69.5
Dibenzo[a,h]anthracene	<200	<200	<500	<500	<1000	20.3	10.6	6.0
Benzo[ghi]perylene	<200	<200	<500	<500	<1000	178.4	121.7	51.9
lndeno[1‚2,3-cd]pyrene	<200	<200	<500	<500	<1000	210.1	51.3	28.0
Phenanthrene, Pyrene, Anthracene, Fluoranthene	<1000 Sum	<5000 Sum	<10,000 Sum	<20,000 Sum	<50,000 Sum	2045.4	426.0	247.1
Naphthalene	<1000	<2000	<2000	<10,000	<10,000	51.5	441.2	54.8
Sum of 15 PAH	<1000	<5000	<10,000	<20,000	<50,000	3810.1	1219.6	512.4

Category 1: Goods to be put in the mouth, or materials in toys, or articles for children up to 3 years old with intended prolonged skin contact (longer than 30 s). Category 2: Goods not covered by category 1 with foreseeable skin contact >30 s (long-term contact), or repeated short-term skin contact. Category 3: Materials not covered in categories 1 or 2, with predictable skin contact up to 30 s (short-term).

**Table 2:** Components of extracted oil [42,43,44].

Retention Time (min)	Components Identification	O70	O80	M.W (g mole−1)	Boiling Point (°C)	GHS Hazard Statements
7.51	Ethylenhexanoic acid	1	2	144.2	228	H361d
8.15	Butoxyethoxy(ethanol)	-	3	162.2	231	H319
8.44	Methoxytriethylenglycol	3	1	164.2	249	Not classified
9.20	Tridecane	-	1	184.4	235	H304
10.10	Tetradecene	1	1	196.3	233	H304, H315
10.17	Tetradecane	1	1	198.4	254	H304
10.73	Butoxytriethylenglycol	1	1	206.3	278	H318
10.99	Tetraethylenglycol monomethylether	3	-	208.3	159	H319
11.03	Pentadecane	-	1	212.4	271	H304
11.106	Di-tert. Butylphenol	1	1	206.3	264	H302, H315, H318, H319, H335, H400 and H410
11.79	Hexadecene	1	1	224.4	285	H304
11.85	Hexadecane	1	1	226.4	287	H304
12.61	Heptadecane	1	1	240.5	302	H304
12.92	Ethylene glycol monobutyl ether	2	-	118.2	171	H302, H311+H331, H315, H319
13.15	Pentaethylen glycol monomethyl ether	2	-	252.3	n. a.	n. a.
13.3	Heptadecanal	1	1	254.5	318	H315 and H319
13.36	Octadecane	1	1	254.5	316	H304
14.05	Nonadecane	1	1	268.5	330	H304
14.15	Di-tert-butyl-1-oxaspiro(4,5)deca-6,9-diene, 2,8-dione	1	2	276.4	515	Not classified
14.44	Hexadecanoic acid	1	1	256.4	352	H315, H319, H335 and H412
14.68	Eicosene	1	1	280.5	341	H304
15.35	Heneicosane	1	1	296.6	359	Not classified
15.71	Octadecanoic acid	1	1	284.5	383	H315, H319, H335 and H412
15.93	Docosene	1	1	308.6	367	H304, H315, H319 and H335
15.96	Docosane	1	1	310.6	370	H315, H319, H335
17.83	Diisooctyl phthalat	1	-	390.6	370	H360 and H413
20.06	Octatriacontanal	1	1	549.0	n. a.	n. a.
20.06	pentafluoropropionate	1	1	163.0	93.50	H315, H319 and H335
22.49	Silane	1	1	32.1	−112	H220 and H280

O70 is the oil extracted at 70 °C and O80 is the oil extracted at 80 °C. 1: low presence, 2: medium presence, 3: high presence and -: no presence.
